# Supplementary material for: New Insights into Phase Separation Processes and Membraneless Condensates of EIN2
Source: Plants (Basel). 2022 Aug 18;11(16):2149. doi: 10.3390/plants11162149 (PMC9416524; doi:10.3390/plants11162149)
Supplement: Supplementary file 1 [file plants-11-02149-s001.zip › plants-1767551-supplementary.pdf]

Supplementary Material

for

New insights into phase separation processes and membrane-less condensates of EIN2

Jian Lu[1], Chi-Kuang Wen[1] and Georg Groth[2]

Author Affiliations:

[1]National Key Laboratory of Plant Molecular Genetics, CAS Center for Excellence in Molecular Plant Sciences, Institute of Plant Physiology and Ecology, Chinese Academy of Sciences, Shanghai 200032, China

[2]Institute of Biochemical Plant Physiology, Heinrich-Heine University Düsseldorf, D-40204 Düsseldorf, Germany

To whom correspondence should be addressed:

Email: georg.groth@hhu.de

Tel: +49 211 81 12822

Fax: +49 211 81 13569

Key words: Liquid-Liquid Phase Separation, Membrane-less Condensates, Ethylene Signaling, EIN2, Intrinsically Disordered Proteins

Supplementary Material - Methods

Rib-seq Methods

Genedenovo Biotechnology Co., Ltd (Guangzhou, China) provided the customer service for constructing the ribosome foot printing libraries, sequencing (on the Illumina NovaSeq 6000 sequencing platform), and data analyses.

References for the involved Ribo-seq analyses service by Genedenovo Biotechnology:

33. Ingolia N T, Ghaemmaghami S, Newman J R S, et al. Genome-wide analysis *in vivo* of translation with nucleotide resolution using ribosome profiling. Science, 2009, 324(5924): 218-223.

34. Ingolia N T, Brar G A, Rouskin S, et al. The ribosome profiling strategy for monitoring translation in vivo by deep sequencing of ribosome-protected mRNA fragments. Nature protocols, 2012, 7(8): 1534.

35. Calviello L, Mukherjee N, Wyler E, et al. Detecting actively translated open reading frames in ribosome profiling data. Nature methods, 2016, 13(2): 165.

36. Zhong Y, Karaletsos T, Drewe P, et al. RiboDiff: detecting changes of mRNA translation efficiency from ribosome footprints. Bioinformatics, 2016, 33(1): 139-141.

The sample preparation, library construction, sequencing, and analyses described below were provided by Genedenovo Biotechnology Co..

Sample preparation

In brief, Arabidopsis seedlings (1 g) were grown on agar (supplemented with 1/2 ×Murashige and Skoog medium) and harvested (in liquid nitrogen) at day 7 (22°C, and 16-hr light/ 8-hr dark). The homogenized samples were centrifuged at 20,000g for 10 min at 4°C, and the supernatant was collected. The supernatant was nest treated with RNase I (NEB, Ipswich, MA, USA) and DNase I (NEB, Ipswich, MA, USA), incubated for 45 min at room temperature and mixed on a Nutator mixer. The nuclease digestion was stopped by adding SUPERase·In RNase inhibitor (Ambion, Austin, TX, USA). Size exclusion columns (illustra MicroSpin S-400 HR Columns; GE Healthcare; catalog no. 27- 5140-01) were equilibrated with 3 mL of polysome buffer by gravity flow and centrifuged at 600 g for 4 min at room temperature. 100µL of digested RFs were added to the column and centrifuged at 600g for 2 min. 10µL 10% (wt/vol) SDS was added to the elution, and RFs with a size greater than 17nt was isolated according to the RNA Clean and Concentrator-25 kit (Zymo Research;R1017). rRNA was removed involving Ribo-Zero rRNA reaction buffer and Ribo-zero Removal solution. Finally, RFs were further purified using magnet beads (Vazyme, Nanjing, Jiangsu, China).

Library construction and sequencing

Ribo-seq libraries were constructed involving NEBNext® Multiple Small RNA Library Prep Set for Illumina® (catalog no.E7300S, E7300L). Briefly, adapters were added to both ends of RFs, followed by reverse transcription and PCR amplification. The 140-160 bp PCR products were enriched to generate a cDNA library and sequenced using Illumina HiSeqTM X10 by Gene Denovo Biotechnology Co. (Guangzhou, China).

Ribo-seq data analysis

Reads filtering

Low quality reads were filtered by fastp [37]. Raw reads containing over 50% of low-quality bases or over 10% of N bases were removed. Adapter sequences were trimmed. Reads with

length between 20-40bp were retained for subsequent analysis. Short reads alignment tool Bowtie2 [38] was used for mapping reads to ribosome RNA (rRNA) database, GenBank, Rfam database. The reads mapped to rRNAs, transfer RNAs (tRNA), small nuclear RNAs (snRNA), small nucleolar RNAs (snoRNA), and miRNA were removed.

Reference genome alignment

Processed RNA-seq reads were aligned to the genome using whole genome alignment by STAR [39] with 2-pass setting enabled. The reads mapped to classical noncoding RNAs (including long noncoding RNAs, microRNA precursors) were abandoned. Then remaining RFs were assigned to different genomic features (5'UTR, CDS, 3'UTR and intron) based on the position of the 5' end of the alignment.

Three nucleotide periodicity analysis

Three-nucleotide periodicity was plotted using the riboWaltz R package [40] as described.

Alignment with Codon distribution analysis

For RFs with specific length (e.g. RFs with length of 30 nt), reads were assigned to frame 1, 2, or 3 according to their position of 5'end in transcripts of protein coding gene, then proportion of total RFs in fame 1, 2 and 3 were calculated among all protein coding genes.

Pause sites analysis

Because the translocation of ribosomes occurs along the mRNA one codon at a time, the potential of single-codon resolution pauses was investigated, involving PausePred (https://pausepred.ucc.ie/).

Quantification of gene abundance

Reads counts in the open reading frame of coding genes was calculated by software RSEM [41], and the gene expression level was normalized by using FPKM (fragment per kilobase of transcript per million mapped reads) method.

Differentially translated genes (DTGs) analysis

To identify differentially translated genes across sample groups, DESeq2 [42] was involved (and by edgeR [43] between two samples). Genes with fold change ≥2 and false discovery rate (FDR) <0.05 in a comparison were considered as significant DTGs.

ORF identification

The longest transcript was selected from alternative splicing of each protein coding gene. A custom ORFfinder search was constructed to search transcript sequence annotated as non-coding region, including 5' untranslated region (UTR), 3'UTR of known protein coding gene. All non-canonical ORFs with length of 60~450 nt (not including stop codons) using start codons ATG were extracted. The non-canonical ORFs included upstream ORF from 5'UTR (uORFs), downstream ORFs from 3'UTR (dORFs).

Translation quantification for ORF

The reads mapped to classical noncoding RNAs (including small nuclear and nucleolar RNAs, microRNA precursors) were abandoned. Then remaining RFs were used to qualify translation level of all ORFs, including main ORF (mORF) of protein coding gene, uORF, and dORF. Reads counts in ORFs was calculated and the translation level was normalized by using FPKM (fragment per kilobase of transcript per million mapped reads) method.

37. Chen, S.; Zhou, Y.; Chen, Y. Fastp: An ultra-fast all-in-one FASTQ preprocessor. *Bioinformatics* 2018, 34, i884–i890

38. Langmead, B.; Salzberg, S.L. Fast gapped-read alignment with Bowtie 2. *Nat. Methods* 2012, 9, 357.

39. Dobin, A. STAR: ultrafast universal RNA-seq aligner. *Bioinformatics* 2013, 29, 15–21

40. Lauria, F.; Tebaldi, T.; Bernabò, P. Ribowaltz: Optimization of ribosome P-site positioning in ribosome profiling data. *PLoS Comput. Biol.* 2018, 14, e1006169

41. Li, B.; Dewey, C.N. RSEM: accurate transcript quantification from RNA-Seq data with or without a reference genome. *BMC Bioinformatics* 2011, 12, 323.

42. Love, M. I.; Huber, W.; Anders, S. Moderated estimation of fold change and dispersion for RNA-seq data with DESeq2. *Genome biology* 2014, 15, 550

43. Robinson, M.D.; McCarthy, D.J.; Smyth, G.K. Edger:aBioconductor package for differential expression analysis of digital gene expression data. *Bioinformatics* 2010, 26, 139–140

Supplementary Material - Figures and Tables

NAME	SIZE	ES	NES	NOM p-val	FDR q-val	FWER p-val	RANK AT MAX
DRLLPS_NUCLEUS_NUCLEOLUS	2154	0.44842	2.30776	0	0	0	7809
DRLLPS_OTHERS_OTHERS	607	0.46411	2.26901	0	0	0	8321
DRLLPS_ALL	3130	0.43382	2.24617	0	0	0	7990
DRLLPS_CLIENT	2438	0.43089	2.21931	0	0	0	7812
DRLLPS_CYTOPLASM_STRESS_GRANULE	541	0.45467	2.19853	0	0	0	6692
DRLLPS_CYTOPLASM_P-BODY	306	0.46812	2.17296	0	0	0	8307
DRLLPS_REGULATOR	363	0.4198	1.99458	0	0	0	8576
DRLLPS_SCAFFOLD	33	0.58446	1.8865	0	0	0	7190
DRLLPS_CYTOPLASM_CENTROSOME/SPINDLE_POLE_BODY	189	0.40441	1.77012	0	0.002276782	0.016	5750
DRLLPS_NUCLEUS_NUCLEAR_SPECKLE	61	0.44401	1.66399	0.001545595	0.00552968	0.044	4892
DRLLPS_NUCLEUS_DNA_DAMAGE_FOCI	5	0.76167	1.4946	0.04109589	0.028586391	0.237	5528
DRLLPS_OTHERS_MITOCHONDRIAL_RNA_GRANUL	15	0.53605	1.44688	0.077319585	0.043524	0.366	6617
PMID31881292_GGRG_MOTIFS	589	0.29461	1.43706	0	0.04427992	0.398	5963
DRLLPS_NUCLEUS_CAJAL_BODY	23	0.43637	1.33696	0.12068965	0.089302644	0.672	7215
DRLLPS_NUCLEUS_HISTONE_LOCUS_BODY	5	0.66939	1.31174	0.13005273	0.10284627	0.749	6681
DRLLPS_CYTOPLASM_SPINDLE_APPARATUS	34	0.40023	1.31155	0.113964684	0.09665086	0.749	5752
PMID31881292_Q/N_RICH_STRECH	532	0.26969	1.30369	0.010146561	0.09904948	0.767	7581
DRLLPS_CYTOPLASM_SIRNA_BODY	4	0.68172	1.23768	0.18914185	0.15359785	0.907	6470
DRLLPS_NUCLEUS_NUCLEAR_PORE_COMPLEX	2	0.80808	1.22701	0.18348624	0.15498757	0.924	4450
DRLLPS_NUCLEUS_PARASPECKLE	6	0.57642	1.19289	0.24875621	0.18329494	0.962	8018
PSPREDICTOR_POTENTIAL_PHASE_SEPARATION_PROTEINS	3857	0.22425	1.1622	0.005	0.21163963	0.981	6500
DRLLPS_NUCLEUS_SAM68_NUCLEAR_BODY	2	0.76416	1.15394	0.27992633	0.21315745	0.985	5468
DRLLPS_CYTOPLASM_PERICENTRIOLAR_MATRIX	1	0.85873	1.13973	0.29681274	0.22373779	0.989	3275
DRLLPS_OTHERS_PYRENOID_MATRIX	36	0.33697	1.12549	0.2852853	0.23573256	0.996	4424
DRLLPS_NUCLEUS_GEMINI_OF_CAJAL_BODY	2	0.67521	1.0118	0.48351648	0.471636	1	7530
DRLLPS_NUCLEUS_PCG_BODY	11	0.26324	0.65846	0.8850772	0.9619016	1	3516

Table S1 Gene sets determined by GSEA involving LLPS. 26 gene sets (GSs) were determined significant (FDR<0.25), and the top 10 up-regulated GSs at the stringent significance level (NOM p<0.01, and FDR q<0.01), for translatome of the wild-type seedlings, compared with that of the *ein2*^{W308*} seedlings. The GS in blue is considered significant at a difference significance level (NOM p<0.05, FDR q<0.05). Size: number of genes in the gene set, ES: enrichment score, NES: normalized ES, NOM p-value: nominal p-values, FWER p-value: familywise error rate.

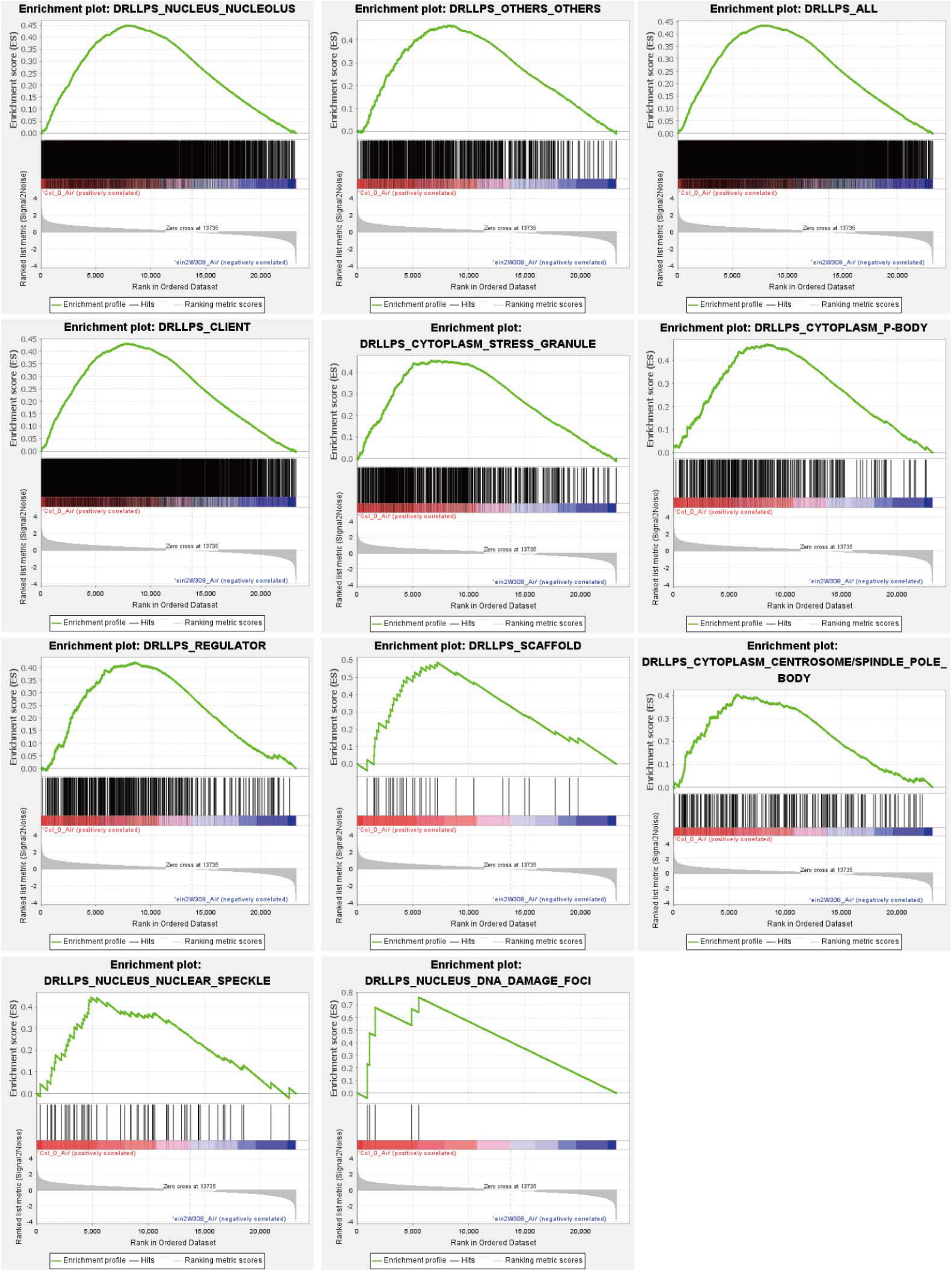

Figure S1. Results of the top 10 gene sets (GSs) for LLPS at the significance level of NOM p<0.01, and FDR q<0.01, and for the GS at the significance level of NOM p<0.05 and FDR q<0.05, respectively.

Supplementary Figure S2: Graphical Quality Indexes for the Ribo-seq Data. Classification of gene reads (A), read length distributions (B), sequencing saturation (C), distribution of ribosome foot printing (RF) (D), and FKPM (Fragments per kilobase of exon model per million mapped fragments) distributions (E) and (F) for the indicated experiments. Meta periodicity plots, involving the read length of the 31-34 bp range, for wild type (Col-0) (G) and ein2W308* (H) samples. In the meta periodicity plot, red for frame 1, green for frame 2, and blue for frame 3. Each genotype involved three independent biological replicates. (F) Data for the green box were not involved in this study.

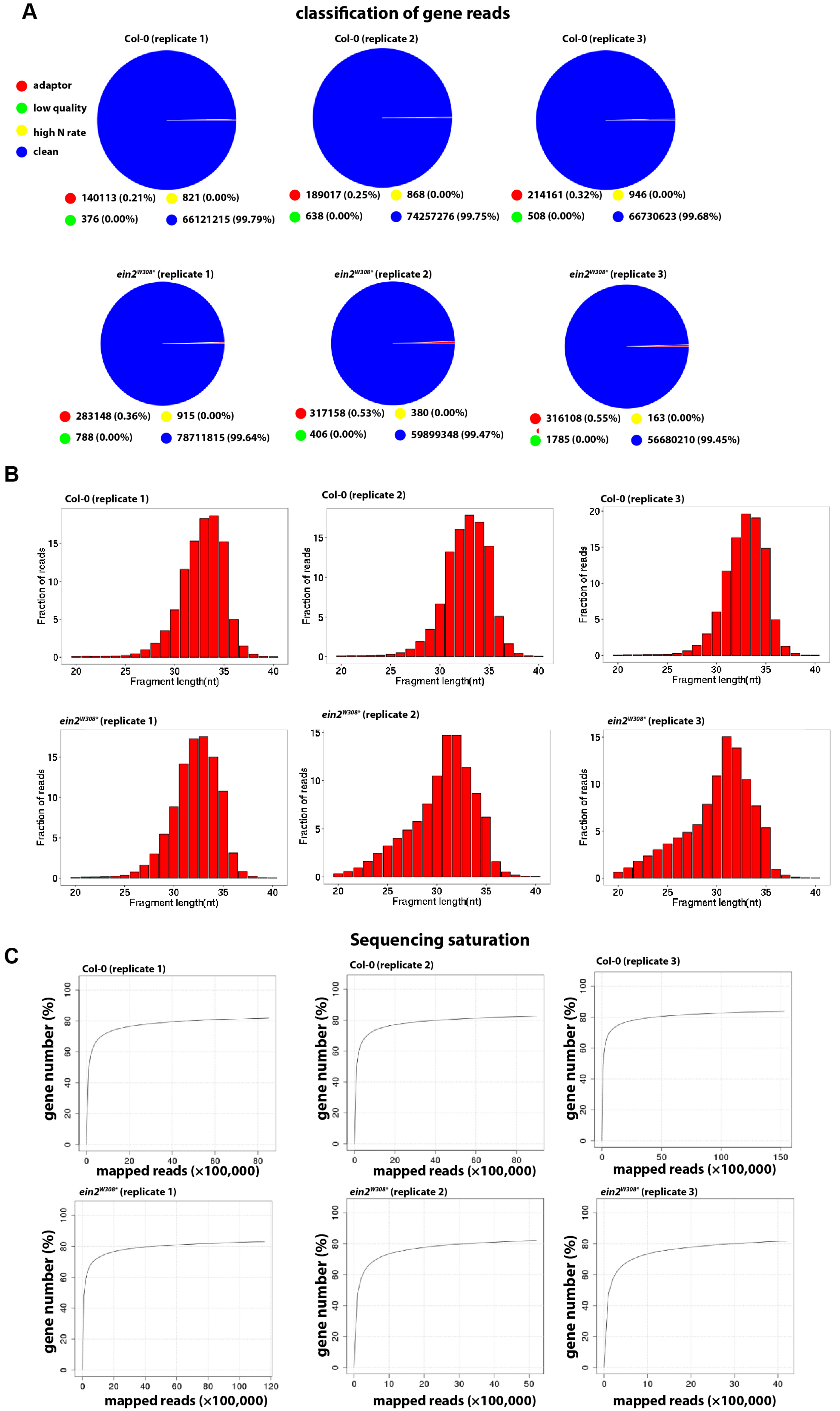
A
classification of gene reads
Col-0 (replicate 1)
Col-0 (replicate 2)
Col-0 (replicate 3)
adaptor
low quality
high N rate
clean
140113 (0.21%)
821 (0.00%)
376 (0.00%)
66121215 (99.79%)
189017 (0.25%)
868 (0.00%)
638 (0.00%)
74257276 (99.75%)
214161 (0.32%)
946 (0.00%)
508 (0.00%)
66730623 (99.68%)
ein2W308* (replicate 1)
ein2W308* (replicate 2)
ein2W308* (replicate 3)
283148 (0.36%)
915 (0.00%)
788 (0.00%)
78711815 (99.64%)
317158 (0.53%)
380 (0.00%)
406 (0.00%)
59899348 (99.47%)
316108 (0.55%)
163 (0.00%)
1785 (0.00%)
56680210 (99.45%)
B
Fraction of reads
Fragment length(nt)
C
Sequencing saturation
gene number (%)
mapped reads (×100,000)

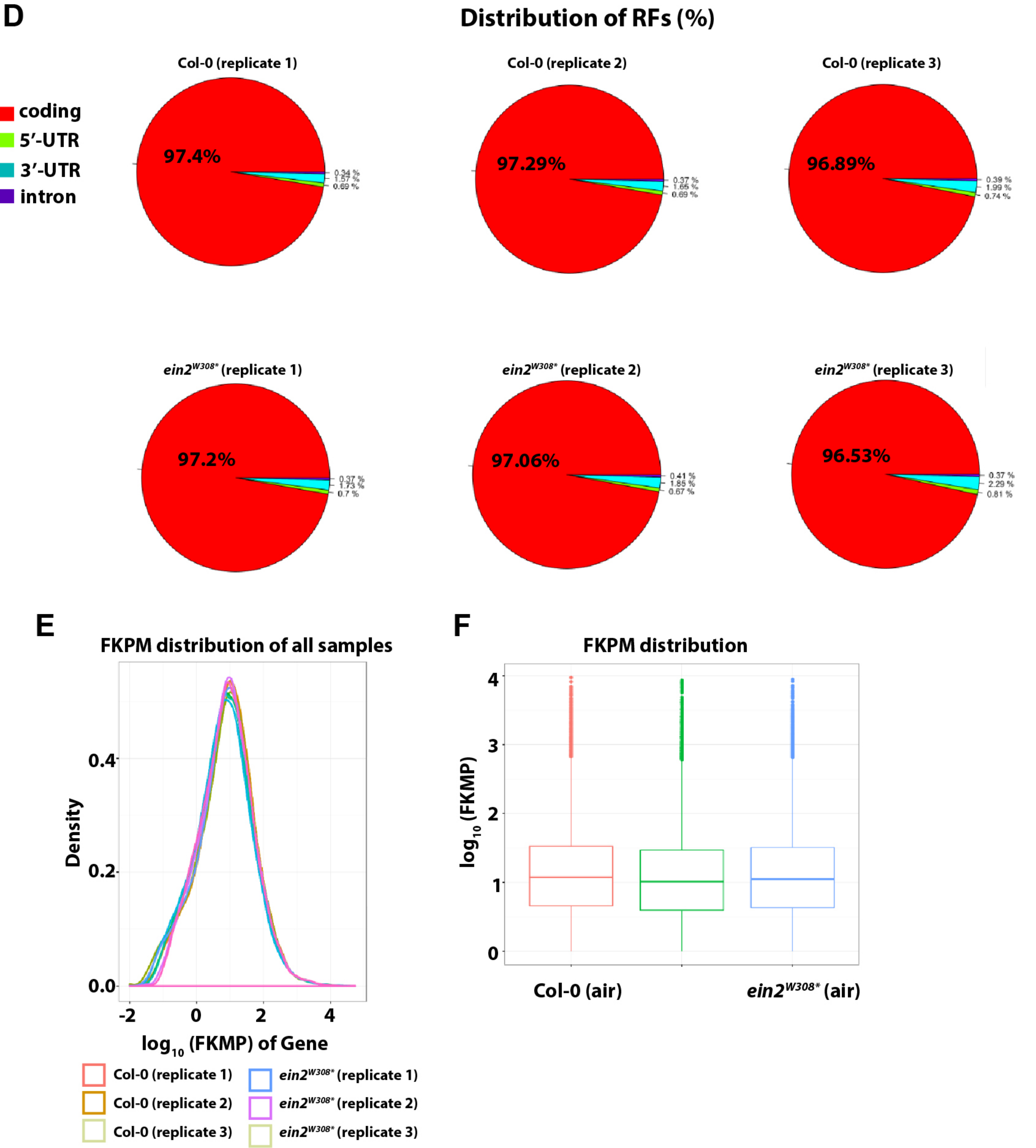

D
Distribution of RFs (%)
Col-0 (replicate 1)
Col-0 (replicate 2)
Col-0 (replicate 3)
coding
5′-UTR
3′-UTR
intron
97.4%
0.34 %
1.57 %
0.69 %
97.29%
0.37 %
1.65 %
0.69 %
96.89%
0.39 %
1.99 %
0.74 %
ein2W308* (replicate 1)
ein2W308* (replicate 2)
ein2W308* (replicate 3)
97.2%
0.37 %
1.73 %
0.7 %
97.06%
0.41 %
1.85 %
0.67 %
96.53%
0.37 %
2.29 %
0.81 %
E
FKPM distribution of all samples
Density
0.4
0.2
0.0
-2
0
2
4
log10 (FKMP) of Gene
Col-0 (replicate 1)
Col-0 (replicate 2)
Col-0 (replicate 3)
ein2W308* (replicate 1)
ein2W308* (replicate 2)
ein2W308* (replicate 3)
F
FKPM distribution
log10 (FKMP)
4
3
2
1
0
Col-0 (air)
ein2W308* (air)

G H

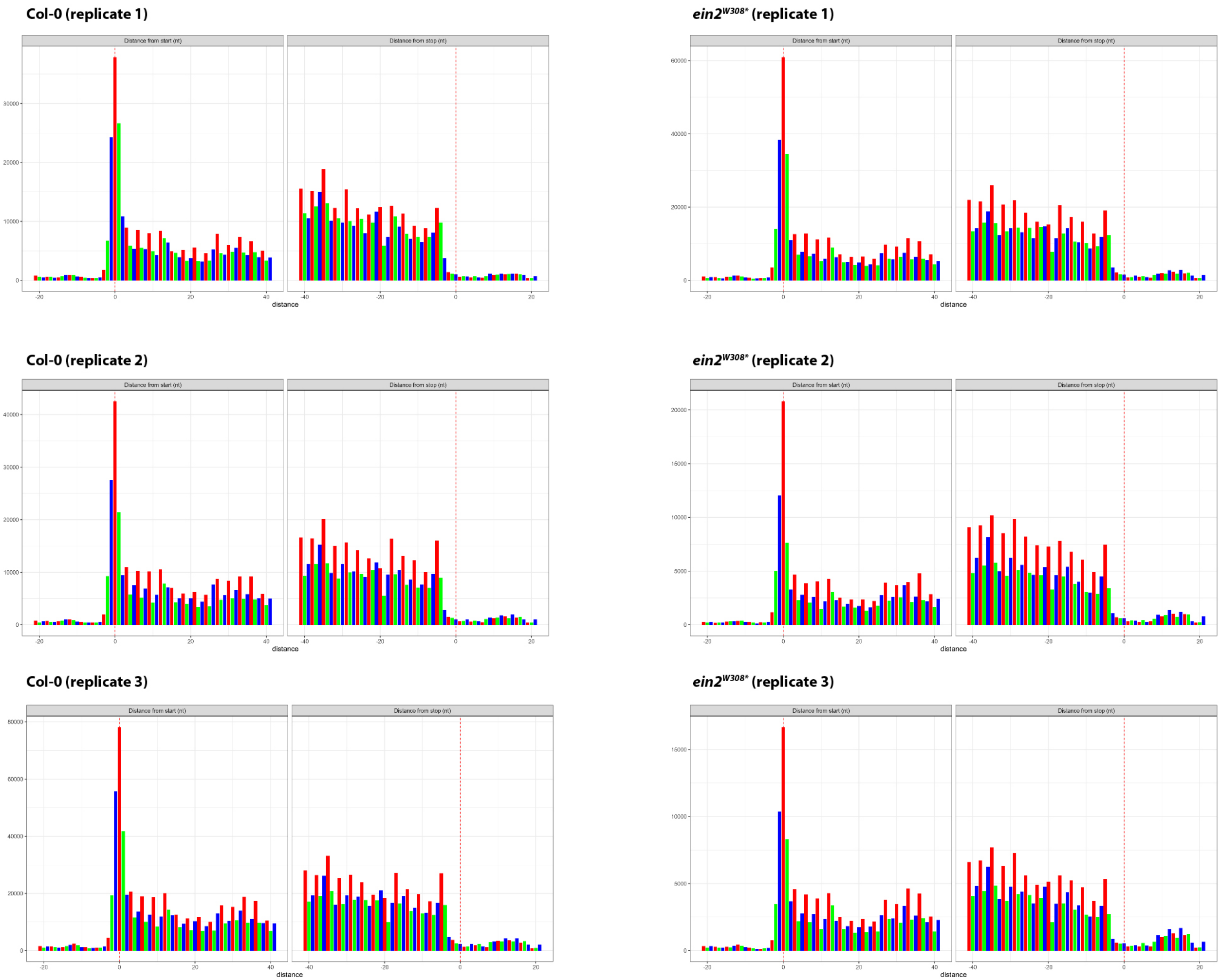
Col-0 (replicate 1)
Col-0 (replicate 2)
Col-0 (replicate 3)
ein2W308* (replicate 1)
ein2W308* (replicate 2)
ein2W308* (replicate 3)
Distance from start (nt)
Distance from stop (nt)
distance

Sample	Reads1 data(bp)	Reads2 data(bp)	Total reads data(bp)
T_Col-0-Air-1	3897675150	3897675150	7795350300
T_Col-0-Air-2	4282184900	4282184900	8564369800
T_Col-0-Air-3	3912450250	3912450250	7824900500
T_ein2W308-Air-1	4508227050	4508227050	9016454100
T_ein2W308-Air-2	3626827450	3626827450	7253654900
T_ein2W308-Air-3	5488561300	5488561300	10977122600

Sample	Before Filter					After Filter				
	Raw Data(bp)	Q20(%)	Q30(%)	N(%)	GC(%)	Clean Data(bp)	Q20(%)	Q30(%)	N(%)	GC(%)
T_Col-0-Air-1	3897675150	3704109365 (95.03%)	3586350231 (92.01%)	944531 (0.02%)	2191349652 (56.22%)	2096511868	2079632140 (99.19%)	2047741869 (97.67%)	929513 (0.04%)	1147753779 (54.75%)
T_Col-0-Air-2	4282184900	4080625477 (95.29%)	3940805591 (92.03%)	775010 (0.02%)	2367907555 (55.30%)	2365049627	2338120551 (98.86%)	2289524043 (96.81%)	757459 (0.03%)	1280524925 (54.14%)
T_Col-0-Air-3	3912450250	3720314534 (95.09%)	3595227914 (91.89%)	962004 (0.02%)	2179864294 (55.72%)	2135173909	2115985243 (99.10%)	2078819743 (97.36%)	944901 (0.04%)	1150818410 (53.90%)
T_ein2W308-Air-1	4508227050	4297163939 (95.32%)	4143279165 (91.90%)	892358 (0.02%)	2511930423 (55.72%)	2457572335	2426752714 (98.75%)	2371755987 (96.51%)	871848 (0.04%)	1356235388 (55.19%)
T_ein2W308-Air-2	3626827450	3421676895 (94.34%)	3293985759 (90.82%)	770424 (0.02%)	2096407966 (57.80%)	1751015514	1735473101 (99.11%)	1705277242 (97.39%)	757522 (0.04%)	1007151291 (57.52%)
T_ein2W308-Air-3	5488561300	4638028074 (84.50%)	4291034425 (78.18%)	520919 (0.01%)	3250594096 (59.22%)	1674932762	1652563733 (98.66%)	1628191135 (97.21%)	492650 (0.03%)	965341766 (57.63%)

Sample	Raw Reads Num	Clean Reads Num(%)	Read Length	Adapter(%)	Low Quality(%)	Poly A(%)	N(%)
T_Col-0-Air-1	66262535	66121215 (99.79%)	150+10	140113 (0.21%)	376 (0%)	10 (0%)	821 (0%)
T_Col-0-Air-2	74447814	74257276 (99.74%)	150+10	189017 (0.25%)	638 (0%)	15 (0%)	868 (0%)
T_Col-0-Air-3	66946251	66730623 (99.68%)	150+10	214161 (0.32%)	508 (0%)	13 (0%)	946 (0%)
T_ein2W308-Air-1	78996687	78711815 (99.64%)	150+10	283148 (0.36%)	788 (0%)	21 (0%)	915 (0%)
T_ein2W308-Air-2	60217317	59899348 (99.47%)	150+10	317158 (0.53%)	406 (0%)	25 (0%)	380 (0%)
T_ein2W308-Air-3	56998290	56680210 (99.44%)	150+10	316108 (0.55%)	1785 (0%)	24 (0%)	163 (0%)

Sample Name	Detected Gene Num
T_Col-0-Air-1	20952 (76.42%)
T_Col-0-Air-2	21145 (77.13%)
T_Col-0-Air-3	21568 (78.67%)
T_ein2W308-Air-1	21268 (77.58%)
T_ein2W308-Air-2	20914 (76.28%)
T_ein2W308-Air-3	20808 (75.90%)
Group Name	Detected Gene Num
T_Col-0-Air	21734 (79.27%)
T_ein2W308-Air	22401 (81.71%)

Table S2. Quality data for the Ribo-seq of wild type (Col-0) and the *ein2*^{W308} mutant.
